# Urban Park Development and Pediatric Obesity Rates: A Quasi-Experiment Using Electronic Health Record Data

**DOI:** 10.3390/ijerph13040411

**Published:** 2016-04-08

**Authors:** TaShauna U. Goldsby, Brandon J. George, Valerie A. Yeager, Bisakha P. Sen, Alva Ferdinand, Devon M. T. Sims, Bryn Manzella, Asheley Cockrell Skinner, David B. Allison, Nir Menachemi

**Affiliations:** 1 Office of Energetics, University of Alabama at Birmingham, Birmingham, AL 35294, USA; brgeorge@uab.edu (B.J.G.); dallison@uab.edu (D.B.A.); 2 Nutrition Obesity Research Center, University of Alabama at Birmingham, Birmingham, AL 35294, USA; 3 Department of Global Health Management and Policy, Tulane University, New Orleans, LA 70112, USA; vayeager@tulane.edu; 4 Department of Health Care Organization and Policy, University of Alabama at Birmingham, Birmingham, AL 35294, USA; bsen@uab.edu; 5 Department of Health Policy and Management, Texas A&M Health Science Center, College Station, TX 77843, USA; ferdinand@tamhsc.edu; 6 Scientific Technologies Corporation, Scottsdale, AZ 85258, USA; devon_sims@stchome.com; 7 Jefferson County Department of Health, Birmingham, AL 35233, USA; bryn.manzella@jcdh.org; 8 Division of General Internal Medicine, The Duke Clinical Research Institute, Duke University, Durham, NC 27705, USA; asheley.skinner@duke.edu; 9 Department of Biostatistics, University of Alabama at Birmingham, Birmingham, AL 35294, USA; 10 Department Health Policy and Management, Indiana University, Indianapolis, IN 46202, USA; nirmena@iu.edu

**Keywords:** childhood obesity, built environment, quasi-experiment, electronic health records

## Abstract

*Introduction*: Childhood obesity affects ~20% of children in the United States. Environmental influences, such as parks, are linked with increased physical activity (PA). *Objective*: To examine whether changes in Body Mass Index (BMI) z-score were associated with construction of a new park. *Methods*: A quasi-experimental design was used to determine whether living in proximity of a park was associated with a reduction in BMI z-score. Children were selected from health clinics within an 11 mile radius of the park. A repeated-measure ANOVA was employed for analysis of the relationship between exposure (new park) and BMI z-score. *Results*: Participants were 1443 (median age 10.3 range (2–17.9 years), BMI: z-score 0.84 ± 1.09) African American (77.4%) adolescents. Change in BMI z-score was not statistically different for children living at different distances from the park after controlling for age, gender, race, ethnicity, or payer type (*p* = 0.4482). We did observe a small 0.03 increase in BMI z-score from pre- to post-park (*p* = 0.0007). There was a significant positive association between child’s baseline age and BMI z-score (*p* < 0.001). *Conclusions*: This study found proximity to a park was not associated with reductions in BMI z-score. Additional efforts to understand the complex relationship between park proximity, access, and PA are warranted.

## 1. Introduction

Childhood obesity affects 17% of children in the United States (U.S.) aged 2 to 19 years [1]. It is widely believed that one way to reduce obesity and promote healthy living among children is through increased physical activity (PA) [2,3,4]. Evidence suggests that built environments, such as sidewalks, parks, trails, and neighborhood safety are associated with increased PA [5,6,7,8,9,10], and several studies have noted that high-quality built environments are associated with increased PA and lower obesity rates while insufficient or low-quality built environments are linked to inadequate levels of PA and increased obesity rates [11,12,13]. Thus, in an effort to combat childhood obesity much policy attention, including a 2012 Institute of Medicine (IOM) report, has been given to establishing or improving local built environments, especially in underserved areas that are more likely to be currently devoid of them [11,13,14].

Recent systematic reviews of the literature concluded that almost all studies examining the built environment’s hypothesized impact on PA or obesity utilized cross-sectional, observational study designs not suitable for establishing causality [15,16,17]. One of the challenges inherent in these studies is the inability to randomly assign individuals to built environments (e.g., those containing or not containing parks). Thus, it is unclear whether people with a proclivity towards healthy living choose to live near such PA-promoting facilities; or whether such facilities, when established, induce PA among people living nearby. Further, it is unclear whether policies encouraging the establishment of new PA facilities will lead to reductions in obesity prevalence.

Quasi-experimental study designs are particularly helpful in “real world” situations where randomization is not feasible. Quasi-experiments allow for a longitudinal observational study, and utilization of empirical techniques, which minimize the effect of confounders, associated with exposure selection. Specifically, quasi-experiments occur when an exogenous event (e.g., a new policy) affects one group (e.g., the exposure group), but not another comparable group (e.g., the control) [18]. The key challenges with such study designs is identifying an exogenous event that affects one group but not another, and having longitudinal data available before and after the event for both groups so as to account for baseline differences between the two groups, as well as temporal changes in the outcomes that occur for both groups. Therefore, differences in outcomes between the exposure and control groups that occur after the exogenous event can, with a reasonable degree of certainty, be causally attributed to the exogenous event [19]. As a result, experts have called for the increased use of such studies for public health decision-making [20,21]. With the increased use of electronic health records (EHR), archived EHR data may provide a useful source of the “before and after” data as exemplified in this study [22].

We utilize the establishment of a new inner-city park as the exposure variable in our quasi-experiment to examine the impact of the park on child obesity rates. We used archived EHR data from six primary care, public health clinics primarily serving low-income, African American children [23]. We consider our exposure group as those children living in closest proximity (*i.e.*, walkable distance) to the newly-established park. Our control group is identified as similar children who live farther away from the park and, thus, presumably cannot walk to the park. Therefore, the purpose of this study was to determine how close to the park is close enough to experience health benefits. We also examine changes in Body Mass Index (BMI) z-scores at various distances from the park.

## 2. Methods

### 2.1. Study Design and Population

Ground breaking for Railroad Park commenced in February 2008, with Railroad Park opening for public use to the inner-city of Birmingham, Alabama in September of 2010. Railroad Park is a 19-acre green space that contains numerous areas for PA for both children and adults. The park includes two age-appropriate play areas with modern playground equipment (e.g., climbing dome, slides, ropes, obstacle courses, and a skateboard park) appropriate for young children and adolescents. Additionally, Railroad Park includes outdoor gym equipment similar to Muscle Beach for outdoor fitness among young adults. Finally, the park includes a large amount of green space and trails for walking and running. The park is open daily from 7 am to 11 pm, and monitored around the clock by a security system and by rangers on patrol. In 2012, the park was the recipient of the Urban Land Institute’s “Urban Open Space Award”. The Urban Space award celebrates and promotes vibrant successful urban open spaces by recognizing spaces that have enriched and revitalized its surrounding community. Awardees receive a plaque and $10,000 dollars to support the organization responsible for the upkeep and/or creation of the winning project. The current study examines how exposure to Railroad Park as measured by the Euclidian distance from one’s home to the park relates to changes in BMI z-scores over time among children.

This study includes a convenience sample of 2151 children under the age of 19 at baseline who were seen in a Jefferson County Department of Health (JCDH) clinic for primary care visits during the study period. Height and weight are routinely taken at every primary care visit and recorded in the JCDH EHR. The study sample was chosen because the availability of the EHR made it possible to calculate BMI z-scores and examine patient demographic data retrospectively and longitudinally.

The study period included a pre-park and post-park time frame. The pre-park time frame included BMI measurements taken 1 February 2009 through 1 September 2010. The post-park time frame included BMI measurement taken 1 March 2011 through 28 February 2012. A lag of six months was selected for the post-park introduction to account for the time necessary to observe BMI z-score changes as a result of any increase in physical activity [24]. To be included in this study, individuals need to be seen at one of the six clinics both pre- and post-park in order to examine the change in BMI z-scores over time. Thus, nine children who were not seen in both the pre-park and post-park time periods were excluded from this study. Additionally, to account for implausibly large changes in BMI (*i.e.*, data recording errors) 671 children whose BMI z-score changed by more than 0.67 between consecutive visits, or by more than 0.33 for visits less than 90 days apart, were excluded from the study [25]. Further, if a family relocated during the study period, the child’s data were excluded from the study; excluding these 28 children ensured that parental preferences for living near a green space were not a source of potential bias for any observed outcomes. After excluding the 708 children from the original sample we were left with 1443 children who met our inclusion criteria.

### 2.2. Variables

Children included in each analysis were divided into two groups: those who live near the park (the exposure group) and those who live far from the park (the control group). For the purpose of this study, nearness was defined as living within 1.5 miles for the exposure group and further than 5 miles for the control group. We also considered two “intermediate” levels of closeness of 1.5 to 3 miles and 3 to 5 miles. The data were extracted from the JCDH EHR and was geocoded using ArcGIS (ArcGIS Desktop: Release 9.0, Environmental Systems Research Institute, Redlands, CA, USA) to measure the Euclidian distance to Railroad Park from the child’s home.

The dependent variable examined in this study was the age and gender specific BMI z-score, a continuous variable. BMI z-score is more appropriate for children than standard BMI. BMI z-scores are measures of relative weight adjusted for child age and sex [26,27]. BMI was also categorized into three groups using the Center for Disease Control and Prevention definitions based on BMI for age percentile growth charts: Normal Weight (5th percentile to less than the 85th percentile), Overweight (85th to less than the 95th percentile), and Obese (equal to or greater than the 95th percentile) [27]. The following variables were included as covariates in the regression model: “age” of the child at the time of the initial visit, gender, race (African American or not), ethnicity (Hispanic or not), and payer type. Payer type was a binary variable denoting self-pay (e.g., paying for services out-of-pocket) *versus* all other means of payment (e.g., Medicaid, CHIP, other type of insurance).

### 2.3. Analyses

All variables were summarized with descriptive statistics. Means and standard deviations were reported to describe the bivariate relationships between exposure (introduction of the park) and the BMI z-score and overweight (85th to less than the 95th percentile) and obese (equal to or greater than the 95th percentile) conditions [27]. The general empirical model used for multivariate analyses utilizes the longitudinal nature of the BMI z-score data to examine the change in BMI z-score among children living closest to the park (the exposure group) compared to the change in BMI z-score among children living farthest from the park (the control group) over the same time period. The effect of the park was modeled as an indicator variable for whether the visit was ”pre-park” or “post-park”. That relationship was analyzed using a repeated-measures ANOVA (RM-ANOVA) with the aforementioned covariates, with a focus on making inference about the interaction between distance category and the “post-park” variable. The temporal correlation was modeled using a compound symmetric correlation structure; due to the irregular time and number of visits, other structures were not considered to be appropriate. For subjects with multiple pre- or post-park visits, all measurements were included in the analysis and denoted as being pre- or post-park.

Additionally, to account for the possibility that the relationship between exposure to the park and BMI may differ for children who were overweight at baseline *versus* not, a second regression model was estimated. It included the baseline weight status for a child (overweight or obese *versus* the reference category of neither) and an interaction term for baseline weight status with distance category and time period (post-park), up to a three-way interaction. Lastly, as a sensitivity analysis we considered an interaction of age with the distance category and the “post-park” variable. Age was considered as a continuous and as a categorical variable (2–6, 6–10, 10–15, 15–19 years old), but was excluded from the final inferential models due to a lack of significance in the two- or three-way interactions (*p* ≥ 0.1218). All data analyses were conducted using SAS Version 9.4 (SAS Institute Inc., Cary, NC, USA). Statistical significance was considered at the *p* < 0.05 level (two-tailed). The study was conducted in accordance with the Declaration of Helsinki and the protocol was approved by the Institutional Review Boards of the JCDH (Protocol No.: N101215006) and the University of Alabama at Birmingham (Protocol No.: E111101001). Compliance approved by the Department of Health and Human Services. This project qualifies as exemption as defined in 45CF46, 101, paragraph. 

## 3. Results

A total of 1443 children were included in this study. The median age for the sample at baseline was 10.3 years, ranging from 2 to 17.9 years (Table 1). Over two-thirds of the sample was African American (77.4%), while White children made up the second largest racial group (21.8%). Based on BMI z-score, some 19.1% of the children were overweight and 25.3% of the children were obese at baseline. The nearest group, which consisted of children living within 1.5 miles of the park, contained 45 children. The intermediate groups (1.5 to 3 miles, 3 to 5 miles) included 164 and 299 children, respectively, while the control group consisted of 935 children living five miles or further from the park.

Descriptive statistics for the BMI z-score for each distance category are given in Table 2. Although the overall mean BMI z-score seem to increase from pre-park to post-park, these are differences are associated with baseline BMI. Specifically, it appears that the children who are overweight or obese at baseline have a smaller increase in BMI z-scores than those who were normal weight; this pattern is consistent with regression to the mean.

In the initial RM-ANOVA, the change in BMI z-scores across the pre-park and post-park periods was not statistically different for children living at different distances from the park after controlling for age, gender, race, ethnicity, or payer type (*p* = 0.4482, Table 3). We did observe a significant increase in BMI z-score from pre- to post-park (*p* = 0.0007). Results for covariates suggested there was a significant positive association between the child’s baseline age and BMI z-score (*p* < 0.0001). Further, being of Hispanic ethnicity was associated with a 0.4 higher BMI z-score (*p* = 0.001).

**Figure 1:**
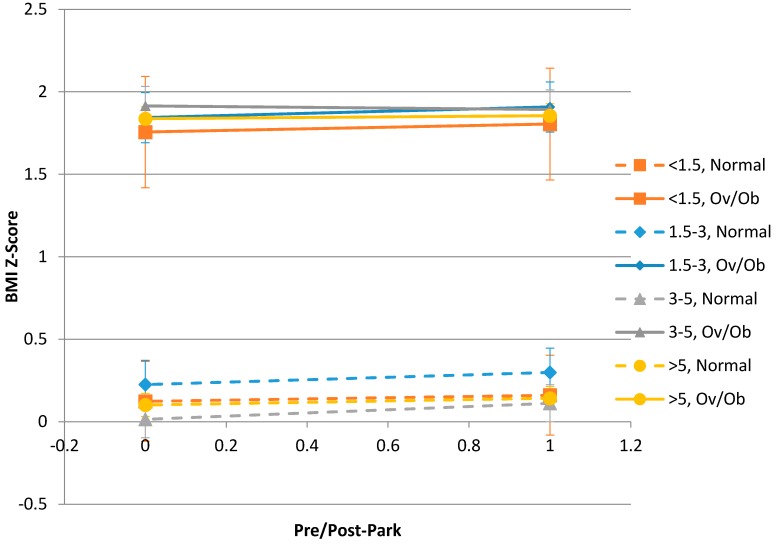
Covariate-adjusted means of BMI z-scores before and after the introduction of the park, by BMI status at baseline (Normal *vs.* Overweight/Obese) and by distance from the park (in miles). The error bars represent 95% confidence intervals around the estimates of the means.

In a second multivariate model that also controlled for baseline overweight status and its interactions with distance and time (Table 4, Figure 1), we did not find a statistically significant difference in the three-way interaction (*p* = 0.0845) for baseline overweight status influencing the association of distance and BMI z-score change over time (which was again not significant, *p* = 0.4804). Children who were overweight or obese at baseline had a 0.02 increase in BMI z-score (95% CI: −0.01, 0.04) while those who were normal weight at baseline had a 0.06 increase (95% CI: 0.03, 0.08), averaged across distance groups; this corresponds to what was seen in Figure 1, although this interaction between time and baseline overweight/obesity status was not significant (*p* = 0.2176). In this model, we saw that while children who were older at baseline still had significantly higher BMI z-score (*p* = 0.0016).

## 4. Discussion

This quasi-experimental study examines the relationship between proximity to a park and BMI z-scores among inner-city children receiving health care in local public health clinics. As a quasi-experiment, this study fills the need for more robust evidence about the relationship between proximity to physical activity-promoting green spaces and obesity at a time when important obesity-related policies and changes are being developed and implemented. It also highlights the feasibility of using archived EHR data for public health policy research. The main findings of this study was the change in BMI z-score across the pre-park and post-park periods was not statistically different for children living at different distances from the park after controlling for age, gender, race, ethnicity, or payer type.

How do we explain why living a certain distance from the park has no statistically significant association with change in BMI z-score? One possible explanation for this finding is that the sample sizes of the near groups were relatively small, potentially limiting the power to identify significant differences between groups. Having more children in the near groups would have been ideal, but being able to examine BMI longitudinally, even in a small group of children, provides valuable information for other obesity researchers and policymakers working to address the U.S. obesity epidemic.

Another explanation for the lack of finding an effect on BMI z-score may be that simply living in a walkable distance to the park may not translate into being more active; other research has noted that children obtain less than 2% of their PA from public parks [28,29]. Further research should continue to examine how close is close enough to experience weight loss and/or health benefits from green spaces. Even if there are increases in PA associated with newly-available parks, it will not lead to reduced obesity-risk if there is a substitution effect (whereby park goers would have used alternative means of PA in the absence of the park) or compensatory increases in caloric intake or decreases in other components of energy expenditure, which largely negate the effects of increased PA. Notably, our study occurred during a time when no concerted efforts were focused on encouraging children to use the park. Future research should focus on maximizing playgrounds and parks as affordable settings to get children to be active, hence, future research might examine how organized children’s activities interact with exposure to the park, or how policies that affect healthy eating interact with exposure to the park in impacting children’s BMI.

There are a number of limitations associated with this study. First, we did not measure proximity to other parks or access to other recreational facilitates. Second, we did not measure park use/visitation or other forms of PA either objectively or subjectively. Although no measure of PA or other health behavior was used or controlled for in this study, it provides an example of a type of methodology that could potentially be useful in future obesity studies or public health policy research. Future interventions should collect data on park use and/or PA as an intermediate outcome to BMI change. The growing utilization of EHRs within and outside the United States may allow for additional, similar longitudinal examinations of changes in obesity among children. Such studies would provide additional robust examinations of the impact of changing obesity-related policy initiatives and interventions. Lastly, while this study examined the impact of the introduction of one urban park in Birmingham, Alabama on children’s BMI z-scores, findings from this study may not be generalizable to other communities, cites, or states.

Despite our non-significant findings, this study is consistent with previous literature that simply changing the built environments is insufficient to promote physical activity [30]. To our knowledge this is the first study investigating obesity-related outcomes of Railroad Park. In addition, this study presents finding on a low-income, traditionally underserved, mostly African American population, which has historically been under studied.

## 5. Conclusions

In conclusion, while the recent U.S. policy attention given to childhood obesity is important, it is vital that researchers continue to examine the impact of obesity-reduction policies so that resources can be used most effectively and efficiently. These findings are important because neighborhood parks provide accessible free spaces for youth to be active [30]. Strategies, such as including community groups to enhance park utilization, should be explored by policy makers. Parks may potentially provide a benefit in terms of child BMI—but simply exposing children to green spaces may not be enough. The use of EHR data could enable future research on the impact of parks and whether efforts conducted to enhance use of parks is effective. Furthermore, EHR data offers a new, more readily available, data source to examine the impact of other health policies (e.g., smoking bans, healthy food choices, *etc.*) on patient-level outcomes [22].

## Figures and Tables

**Table 1:** Sample descriptive characteristics at baseline.

Characteristics	Total Sample *N* = 1443	Near (<1.5 miles) *N* = 45	Intermediate (1.5–3 miles) *N* = 164	Intermediate (3–5 miles) *N* = 299	Control (>5 miles) *N* = 935
Median age (range)	10.3 (2, 17.9)	9 (2, 16.9)	10.5 (2, 17.7)	10.8 (2, 17.6)	10.3 (2, 17.9)
Race					
Black	1117 (77%)	35 (78%)	140 (85%)	270 (90%)	672 (72%)
White	315 (22%)	10 (22%)	21 (13%)	27 (9%)	257 (27%)
Other	11 (1%)	0 (0%)	3 (2%)	2 (1%)	6 (1%)
Ethnicity					
Hispanic	196 (14%)	8 (18%)	22 (13%)	29 (10%)	137 (15%)
Non-Hispanic	(1247 (86%)	37 (82%)	142 (87%)	270 (90%)	798 (85%)
Gender					
Female	809 (56%)	26 (58%)	91 (55%)	160 (54%)	532 (57%)
Male	634 (44%)	19 (42%)	73 (45%)	139 (46%)	403 (43%)
Overweight or Obese					
Overweight (85%–95%)	276 (19%)	7 (16%)	34 (21%)	53 (18%)	182 (19%)
Obese	365 (25%)	8 (18%)	44 (27%)	81 (27%)	232 (25%)
Payer type					
Self-pay	570 (40%)	23 (51%)	69 (42%)	100 (33%)	378 (40%)
Other	873 (60%)	22 (49%)	95 (58%)	199 (67%)	557 (60%)

**Table 2:** Changes in BMI z-scores among children living at various distances from the park.

Distance from Park	*N*	Mean (SD) BMI z-Score		
		First Visit (Pre-Park)	Final Visit (Post-Park)	Change (Final-First)
All Children	1443	0.84 (1.09)	0.88 (1.10)	0.04 (0.33)
Near (within 1.5 miles)	45	0.61 (1.00)	0.66 (1.09)	0.05 (0.33)
Intermediate (Between 1.5 and 3 miles)	164	0.96 (1.00)	1.03 (1.04)	0.07 (0.31)
Intermediate (Between 3 and 5 miles)	299	0.83 (1.15)	0.87 (1.12)	0.04 (0.31)
Control Group (5 miles or more)	935	0.83 (1.09)	0.87 (1.11)	0.03 (0.33)
Normal Weight Children at Baseline (BMI z-score < 85)	802	0.05 (0.73)	0.11 (0.78)	0.06 (0.35)
Near (within 1.5 miles)	30	0.06 (0.70)	0.11 (0.86)	0.05 (0.36)
Intermediate (Between 1.5 and 3 miles)	86	0.18 (0.63)	0.25 (0.71)	0.07 (0.35)
Intermediate (Between 3 and 5 miles)	165	−0.03 (0.70)	0.06 (0.73)	0.09 (0.33)
Control Group (5 miles or more)	521	0.06 (0.75)	0.10 (0.80)	0.04 (0.36)
Overweight/Obese Children at Baseline (BMI z-score ≥ 85)	641	1.82 (0.52)	1.84 (0.57)	0.02 (0.29)
Near (within 1.5 miles)	15	1.71 (0.41)	1.77 (0.51)	0.06 (0.27)
Intermediate (Between 1.5 and 3 miles)	78	1.81 (0.49)	1.88 (0.55)	0.07 (0.27)
Intermediate (Between 3 and 5 miles)	134	1.89 (0.56)	1.86 (0.59)	−0.02 (0.26)
Control Group (5 miles or more)	414	1.81 (0.51)	1.83 (0.57)	0.02 (0.30)

**Table 3:** Relationship between the interaction between the park and being near to the park and BMI z-scores compared to children living far from the park (>5 miles).

Variable	Regression Coefficient	SE	Type III *p*-Value
Near (within 1.5 miles)	−0.1895	0.1656	0.2519
Intermediate (Between 1.5 and 3 miles)	0.1243	0.0924	
Intermediate (Between 3 and 5 miles)	0.0079	0.0733	
Post-park	0.0302	0.0098	0.0007
Near × Post-park	0.0107	0.0460	0.4482
(1.5–3 miles) × Post-park	0.0401	0.0254	
(3–5 miles) × Post-park	0.0136	0.0199	
Age at baseline	0.0027	0.0005	<0.0001
Self-pay	−0.0115	0.0108	0.2895
Male	−0.0366	0.0574	0.5233
Black	0.0502	0.0961	0.6019
Hispanic	0.4506	0.1168	0.0001

**Table 4:** Relationship between the interaction between the park, being near to the park, and BMI z-scores controlling for baseline BMI compared to children living far from the park.

Variable	Regression Coefficient	SE	Type III *p*-Value
Near (within 1.5 miles)	0.0221	0.1252	0.4626
Intermediate (Between 1.5 and 3 miles)	0.1239	0.0776	
Intermediate (Between 3 and 5 miles)	−0.0865	0.0600	
Post-park	0.0406	0.0132	0.0015
Overweight/obese at baseline	1.7354	0.0439	<0.0001
Near × Post-park	−0.0033	0.0572	0.4804
(1.5–3 miles) × Post-park	0.0330	0.0345	
(3–5 miles) × Post-park	0.0561	0.0266	
Near × Ovw/ob at baseline	−0.1032	0.2145	0.3002
(1.5–3 miles) × Ovw/ob at baseline	−0.1173	0.1127	
(3–5 miles) × Ovw/ob at baseline	0.1640	0.0887	
Ovw/ob at baseline × Post-park	−0.0224	0.0196	0.2176
Near × Post-park × Ovw/ob at baseline	0.0335	0.0912	0.0845
(1.5–3 miles) × Post-park × Ovw/ob at baseline	0.0134	0.0508	
(3–5 miles) × Post-park × Ovw/ob at baseline	−0.0961	0.0399	
Age at baseline	0.0010	0.0003	0.0016
Self-pay	−0.0115	0.0107	0.2828
Male	0.0039	0.0348	0.9103
Black	0.0116	0.0581	0.8417
Hispanic	0.1098	0.0710	0.1221
